# Quantitative Competition Binding of Fluorinated Ligands by Real‐Time ^19^F In‐Cell NMR to Assess Isoform Selectivity in Human Cells

**DOI:** 10.1002/mrc.70098

**Published:** 2026-03-22

**Authors:** Azzurra Costantino, Letizia Barbieri, Simone Giovannuzzi, Alessio Nocentini, Claudiu T. Supuran, Enrico Luchinat

**Affiliations:** ^1^ Magnetic Resonance Center—CERM University of Florence Sesto Fiorentino Italy; ^2^ Interuniversity Consortium for Magnetic Resonance of Metalloproteins—CIRMMP Sesto Fiorentino Italy; ^3^ NEUROFARBA Department, Section of Pharmaceutical and Nutraceutical Sciences University of Florence Sesto Fiorentino Italy; ^4^ Chemistry Department University of Florence Sesto Fiorentino Italy

## Abstract

Drug development is a risky endeavour with a high failure rate, often caused by the limited ability to predict the efficacy and interactions of candidate drugs in a native cellular environment. In this context, in‐cell NMR spectroscopy is a promising tool for assessing drug‐target binding directly in living cells, thereby improving the screening and development of new molecules. In this study, we used real‐time in‐cell ^19^F NMR spectroscopy in a flow bioreactor to observe competitive binding of fluorinated benzenesulfonamide derivatives to three cytosolic isoforms of carbonic anhydrase. Quantitative measurement of the dissociation constants relative to a spy ligand allowed an accurate ranking of the compounds based on their intracellular affinities for each isoform. The use of two fluorinated ligands allowed simultaneous observation of spy ligand displacement and test ligand binding, as well as estimation of the effective ratio of free ligand concentrations under poor solubility conditions. We also show that signal saturation caused by short repetition times, which can significantly impact the analysis, can be easily corrected a posteriori. Overall, we show that real‐time in‐cell ^19^F NMR spectroscopy can reliably quantify drug–target binding in the cellular environment, paving the way for future applications in drug discovery.

## Graphical abstract

Real‐time in‐cell ^19^F NMR spectroscopy in a flow NMR bioreactor was used to monitor the binding of fluorinated compounds to three cytosolic carbonic anhydrase isoforms in living human cells. The simultaneous observation of the two ligands allowed for the quantitative ranking of their intracellular affinities by competition binding. This approach allows for screening drug candidates directly in the native cellular environment, making it a promising tool in early drug discovery.

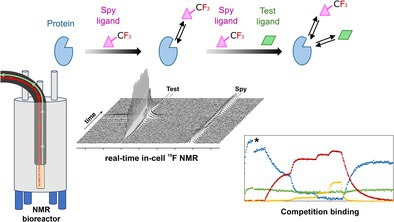

## 1 Introduction

Development of new drugs is a long and expensive process, with only a limited number of drug candidates achieving regulatory approval each year. Many promising candidate drugs do not make it through the preclinical tests, and some even fail during clinical trials due to low efficacy, emerging toxic effects or poor pharmacological properties, thus making drug development a highly risky endeavour [1, 2]. This high attrition rate outlines the need for more effective predictive tools to better evaluate drug molecules in the early stages of drug discovery process, in a physiologically relevant setting. An important limitation of current preclinical pipelines is that screening and optimization of novel compounds are typically performed in vitro, without taking into account the complexity of the cellular milieu. Indeed, the subcellular localization of the target protein, as well as its interactions with the environment, can greatly influence the pharmacological properties of small molecules. Consequently, drug candidates that appear promising in vitro may not actually engage their intracellular target in vivo.

In this context, in‐cell NMR spectroscopy has the potential to bridge the gap between traditional in vitro studies and in vivo models. When observing intracellular targets, in‐cell NMR enables the investigation of macromolecular structure, dynamics and interactions with cellular partners or exogenous ligands within living cells [3, 4, 5]. This approach can be applied during early‐stage drug evaluation, particularly in the optimization phase of lead compounds, when a limited number of candidate compounds require characterization of target engagement under near‐physiological conditions [6, 7]. Alternatively, small molecules can be selectively observed as they interact with the target by exploiting the presence of fluorine atoms [8, 9, 10]. ^19^F NMR is advantageous for in‐cell studies as it is sensitive, reduces spectral complexity and is less affected by the slow tumbling of the target, as internal motions of the bound ligand lead to favourable spin relaxation properties [11, 12, 13, 14].

When investigating ligand binding affinity by direct titration, NMR spectroscopy is typically limited to dissociation constants (*K*_*d*_) in the μM–mM range. For compounds binding with higher affinity, such as those found in the hit‐to‐lead optimization phase of drug development, competitive binding can be employed [15, 16]. In this experiment, a reference compound, the ‘spy’ ligand, initially bound to the target, is progressively displaced by a second ‘test’ ligand. The displacement curve can then be fitted to obtain the *K*_*d*_ of the test ligand. Conducting such experiments on cell pellets sedimented in the NMR tube would require multiple independent samples, due to the limited sample lifetime, thus increasing the overall sample preparation and acquisition time. Alternatively, a flow NMR bioreactor can be employed [17, 18, 19]. In this setup, cells are kept alive and in a metabolically active state through a continuous flow of fresh medium to provide fresh nutrients and remove metabolic byproducts, thereby enabling prolonged monitoring of intracellular processes by NMR spectroscopy. With the NMR bioreactor, competitive binding studies can be performed on a single sample of cells by real‐time in‐cell NMR analysis. This approach has been recently applied to measure ligand binding affinity towards an intracellular target protein, both by protein‐ and ligand‐observed in‐cell NMR [14, 20]. In these experiments, a spy ligand is continuously provided to the sample at a constant concentration throughout the experiment, whereas the test ligand is introduced stepwise at increasing concentrations. In the protein‐observed mode, competitive binding is monitored from the signals of the target protein in complex with either the spy or the test ligand, which are in slow exchange [20]. In the ligand‐observed mode, competitive binding is monitored by the decreasing ^19^F signal of the spy ligand in complex with the target [14]. Provided the intracellular affinity of the spy ligand is known, this approach allows quantitative measurements of intracellular *K*_*d*_ in the nanomolar range.

In this study, we show that the above approach can be used for affinity ranking of fluorinated lead compounds towards different isoforms of the intracellular target. To this aim, we applied real‐time in‐cell ^19^F NMR spectroscopy to quantitatively investigate the competition between a set of recently reported fluorinated benzenesulfonamide derivatives targeting carbonic anhydrase (CA) [21]. CA is a ubiquitous metalloenzyme that catalyses the reversible hydration of CO_2_ in the organism and is inhibited by benzenesulfonamides, a class of small‐molecule inhibitors that coordinate the active site zinc ion. We focused on three cytosolic isoforms, CA1, CA2 and CA13, which share a highly conserved active site. CA1 is primarily expressed in erythrocytes, muscle and various organs; CA2 is ubiquitously distributed; and CA13 is found mainly in the small intestine, prostate and colon. Using one of the fluorinated ligands as a spy in competition against each of the other fluorinated ligands and correlating the spy ligand with a known reference inhibitor, we determined the intracellular *K*_*d*_ for each ligand relative to the reference inhibitor, thus providing quantitative rankings of the compounds for each CA isoform.

## 2 Materials and Methods

### 2.1 Ligand Stability Assessed by ^19^F NMR Spectroscopy

Fluorinated compounds were dissolved in DMSO to a concentration of 80 mM. To evaluate their stability in cell culture medium, each compound was diluted to a final concentration of 100 μM in high‐glucose Dulbecco's Modified Eagle Medium (DMEM, Gibco) supplemented with 2% foetal bovine serum (FBS, Gibco), 1% penicillin/streptomycin (100× solution, Gibco) and 3% D_2_O. Sample stability was assessed by ^19^F NMR spectroscopy immediately after preparation and after 72 h of incubation at 37°C. ^19^F NMR spectra were acquired at 310 K at a 600‐MHz Bruker Avance NEO spectrometer equipped with a QCI‐F 5‐mm Cryoprobe operating at 564.6 MHz. A single 90° pulse was used (zg Bruker pulse programme), with a 1.2‐s acquisition time, a frequency offset of −66.0 ppm and a spectral width of 49.2 ppm. Each spectrum was recorded with 32 scans and a 10‐s interscan delay, resulting in a total acquisition time of approximately 7 min per sample.

### 2.2 Human Cell Culture and Transfection

HEK293T cells (ATCC CRL‐3216) were cultured in uncoated 75‐cm^2^ flasks using high‐glucose DMEM, supplemented with 10% FBS, L‐glutamine (100× solution, Gibco) and 1% penicillin/streptomycin. Cells were maintained at 37°C in a humidified incubator with 5% CO_2_. For transient expression of carbonic anhydrase isoforms, cells were transfected with pHL‐CA1, pHL‐CA2 or pHL‐CA13 plasmids obtained previously [21] using polyethylenimine (PEI) at a DNA:PEI mass ratio of 1:2 (25‐μg DNA, 50‐μg PEI), according to a previously published protocol [22]. The expression level of each protein was assessed by SDS‐PAGE and was consistent with what was previously reported using the same protocol [21].

### 2.3 Cell Encapsulation in Agarose Threads

Cells were encapsulated in agarose threads as previously reported [23]. Low‐gelling temperature agarose (Sigma−Aldrich) was prepared at 1.5% (w/v) in phosphate‐buffered saline (PBS) by heating to 85°C, sterilized by filtration and stored at 4°C until use. For cell encapsulation, an aliquot of the agarose solution was melted at 85°C and maintained at 37°C. Cells harvested from a single 75‐cm^2^ flask were prewarmed at 37°C and resuspended in 450 μL of agarose solution. The resulting cell‐agarose suspension was drawn into PEEK chromatography tubing (inner diameter, i.d., 0.75 mm) connected to a 1‐mL syringe and allowed to cool at room temperature for 2 min to solidify. The resulting agarose threads were then transferred into the flow NMR tube, prefilled with PBS.

### 2.4 NMR Bioreactor Setup and Operation

**FIGURE 1:**
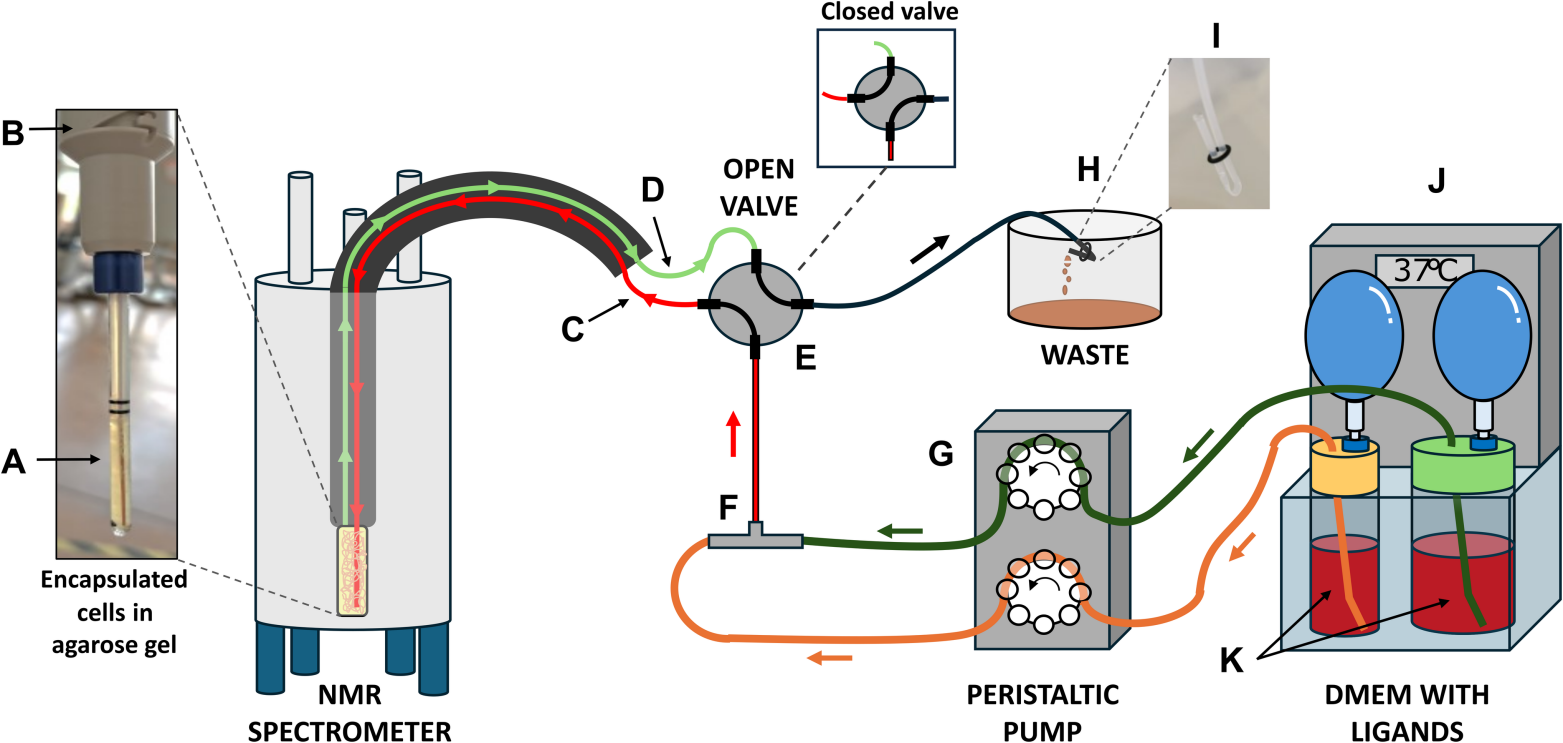
Experimental setup of the NMR bioreactor. (A) Flow NMR tube filled with encapsulated cells; (B) spinner‐shaped end of the flow unit; (C) PEEK tubing inlet; (D) PTFE tubing outlet; (E) four‐way valve; (F) PEEK tee connector; (G) multichannel peristaltic pump; (H) waste container; (I) flow restrictor; (J) circulating water bath; (K) medium reservoirs. [Colour figure can be viewed at wileyonlinelibrary.com]

The NMR bioreactor was assembled similarly to previously described [23]. Briefly, the 5‐mm flow NMR tube containing the encapsulated cells (Figure 1A) was connected to the tube holder of the flow unit (InsightMR 2.0, Bruker) (Figure 1B). A PEEK tubing inlet (o.d. 1/32″, i.d. 0.5 mm) (Figure 1C) was inserted in the sample tube, whereas a polytetrafluoroethylene (PTFE) tubing outlet (o.d., 1/16″, i.d. 1 mm) (Figure 1D) was connected at the top of the tube holder. Compared with the flow unit employed previously (InsightMR 1.0, Bruker), the device used here has a wider exit channel at the top of the flow tube, which may cause sample loss. To prevent this, a 4‐mm diameter circular‐shaped mesh was cut from a 100‐μm mesh cell strainer (Corning), pierced with a needle and slid along the PEEK inlet tubing up to the entrance of the exit channel, to confine the agarose gel in the flow tube while allowing liquid and small debris to pass through without clogging. The bioreactor was maintained at 37°C using a circulating water bath (Julabo) (Figure 1J). The inlet and outlet tubing were connected through a four‐way valve (Figure 1E) to the pump (Figure 1G) and to the waste container (Figure 1H). Cells were perfused with unlabelled high‐glucose DMEM (Sigma‐Aldrich; powder, reconstituted in sterile‐filtered Milli‐Q water) supplemented with 2% FBS, 10‐mM NaHCO_3_, 1% penicillin/streptomycin, 3% D_2_O and one or two fluorinated compounds at defined concentration, adjusted to pH 7.4. Using two channels of a multichannel peristaltic pump (RegloICC, Ismatech), the flow from two medium reservoirs (Figure 1K) was merged upstream of the valve using a PEEK tee connector (Figure 1F). The culture media were stored in 250‐ or 500‐mL glass reservoirs and kept at 37°C in the water bath throughout the experiment. The flow rates of the two channels were varied via the pump software to change the compound concentrations in a stepwise manner, keeping the total flow rate constant at 0.1 mL/min. The compound concentrations and flow profiles used at each step of the experiment are reported in Table S1. Compared with previous configurations, the bioreactor setup was modified to provide constant internal pressure throughout the system in order to minimize gas bubble formation. Specifically, a flow restrictor (Figure 1I) was created by pinching the PTFE tubing at the end of the circuit, before the waste container, and two air‐filled balloons were attached to each filter in the bottles containing reconstituted DMEM. These balloons apply a slight positive pressure to the reservoirs, preventing the spontaneous release of dissolved CO_2_.

### 2.5 Time‐Resolved In‐Cell ^19^F NMR Analysis

Time‐resolved ^19^F NMR spectra were acquired at 310 K at a 600‐MHz Bruker Avance NEO spectrometer equipped with a QCI‐F 5‐mm Cryoprobe operating at 564.6 MHz. The ^19^F chemical shift scale was calibrated by setting the signal of trifluoroacetic acid in an external reference sample to −76.55 ppm (with respect to trichlorofluoromethane). A single 90° pulse was employed, followed by FID acquisition (zg Bruker pulse programme) with a frequency offset of −54.3 ppm and a spectral window of 50.3 ppm. For time‐resolved in‐cell NMR experiments recorded in the bioreactor, a series of ^19^F NMR spectra was acquired with 512 scans and a 1‐s recycle delay, yielding a time resolution of 11 min per spectrum over a total acquisition time of up to 66 h. The resulting spectral series were processed with Topspin (Bruker) and analysed using the Dynamics Center software (Bruker), where peak integrals were measured as a function of time.

The fraction of intracellular CA bound to each ligand was quantified by measuring the peak area of the corresponding CA–ligand complex at equilibrium, obtained by averaging over a 1‐h period for each step of the run after reaching the plateau values. In the case of compounds that give rise to an additional broad signal in cells [21], the correct peak areas were obtained by either applying baseline correction or by subtracting an adjacent same‐width integration area equally affected by the broad signal. The peak areas were then corrected to account for signal saturation (vide infra). Because 100% of the intracellular CA is bound to either of the two ligands at any moment of the run, the fraction of CA bound to the spy ligand was calculated by dividing the averaged peak area of the CA–spy complex by the sum of the averaged peak areas of both the CA–spy and CA–test complexes when both peaks were visible (bioreactor runs 1–9). In bioreactor runs 10–12, where only the CA–spy complex peak was detected, the total protein concentration was estimated as previously reported [14], that is, by fitting the signal intensity between the final point of the first step, where all CA was bound to the spy ligand, and the point of the wash step where the CA–spy complex was fully restored. The obtained total protein concentration at each time point was then used to calculate the fraction of CA bound to the spy ligand throughout the run. For bioreactor runs 1–9, the ratio of the concentrations of free test and free spy ligand in the flow tube was calculated from the peak areas of the corresponding signals at equilibrium, averaged over a 1‐h plateau period for each step. However, in some competition binding experiments, the peak area of the free test ligand was not measurable due to overlap with the corresponding CA–ligand complex peak, such as in bioreactor run 1, where compounds **6** and **4** competed for binding to the CA1 protein. Since the free ligand ratios were found to be consistent across different runs with the same two ligands, the ratios from another run were used whenever signal overlap prevented correct signal integration. Error bars were calculated by standard error propagation from the standard deviations of the corresponding mean peak areas.

### 2.6 Curve Fitting

Nonlinear curve fitting was performed using OriginPro 8 (OriginLab) to estimate the dissociation constant of each test ligand relative to that of the spy ligand. The fitted data points correspond to the fraction of intracellular CA bound to the spy ligand (*F*_*S*_) at a defined ratio of test (*T*) and spy (*S*) ligand concentrations. *K*_*d*_ ratios were obtained by fitting the binding curves with the previously described equation: [20, 24]

(1) $$F_{S}=\frac{1}{1+\frac{K_{\mathrm{dS}}\left[T\right]}{K_{\mathrm{dT}}\left[S\right]}}$$

where *F*_*S*_ is the fraction of CA isoform bound to *S*, [*T*]/[*S*] is the ratio of the concentrations of free *T* and *S*, and *K*_*dT*_/*K*_*dS*_ is the ratio of the dissociation constants of *T* and *S*.

In the NMR bioreactor setup, direct quantification of intracellular free ligand concentrations cannot be obtained, due to the low filling factor of the cells in the flow tube and, likely, due to exchange broadening from weak interactions with the cellular milieu. However, at equilibrium, it is assumed that for passively diffusing compounds, such as neutral sulfonamides [25], the intracellular concentrations of free ligands are equal to those in the external solution, according to the free drug hypothesis [26], as both ligands are supplied in excess by the continuously perfused medium and are able to saturate all intracellular binding sites. Therefore, [*S*] and [*T*] are taken equal to the concentrations of the ligands in the perfusion medium.

### 2.7 *T*_1_ Measurement and Saturation Factor (SF) Calculation

Longitudinal relaxation times (*T*_1_) of the CA2–ligand complexes were measured by inversion recovery on HEK293T cells transiently transfected with CA2 and treated with compounds **4**, **5**, **6** and **7**. A series of ^19^F NMR spectra was acquired using variable recovery delays of 0.01, 0.1, 0.25, 0.5, 0.75, 1, 2 and 5 s. The acquired spectra were analysed using the Dynamics Center software (Bruker), and *T*_1_ values were extracted by fitting the plot of the peak area of the CA2–ligand complex as a function of the delay time. The fitting procedure also provided the corresponding standard error for each *T*_1_ value.

From the measured *T*_1_ values, the SF was calculated according to Equation (2), which describes the ratio between the partially saturated and fully relaxed signal amplitudes, as previously reported [27].

(2) $$\mathrm{SF}\left(\theta ,T,T_{1}\right)=\frac{\left(1-E_{1}\right)\sin\theta }{1-E_{1}\cos\theta }\;\text{where}\;E_{1}=\exp\left(-\frac{T}{T_{1}}\right)$$

Here, *θ* is the flip angle (90° in this case), *T* is the repetition time, and *T*_1_ is the longitudinal relaxation time.

Once determined using Equation (2), the SF was used as a correction factor to retrieve the fully relaxed (unsaturated) signal amplitudes from the experimentally observed signals, which are partially saturated due to the use of short repetition times:

(3) $$M_{0}=M_{f}\cdot \hat{y}/\mathrm{SF}\left(\theta ,T,T_{1}\right)$$

where *M*_0_ denotes the equilibrium magnetization along the static magnetic field, *M*_*f*_ the magnetization vector following a flip during steady state and $\hat{y}$ denotes the unit vector along the *y*‐axis.

## 3 Results and Discussion

**Chart 1:**
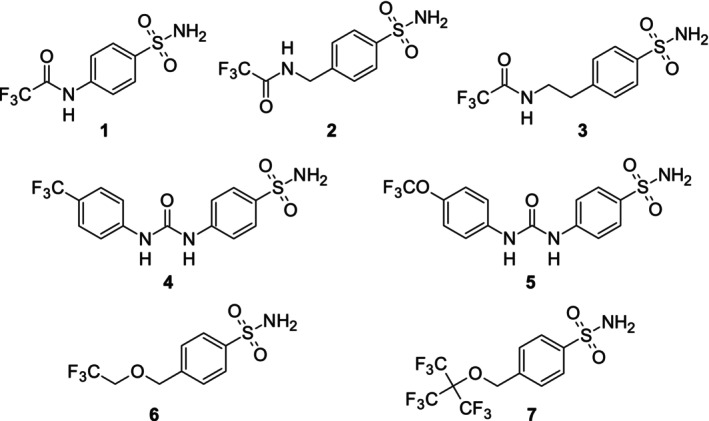
Fluorinated benzenesulfonamide derivatives used in this study.

A series of fluorinated CA inhibitors (Chart 1) previously characterized [21] were used as ligands for competitive binding targeting different CA isoforms. These compounds share a benzenesulfonamide scaffold and contain one (**1**–**6**) or three equivalent (**7**) trifluoromethyl groups, allowing detection by ^19^F NMR spectroscopy.

To evaluate the suitability of the ligands for competition binding assays by real‐time in‐cell NMR, we assessed their stability in DMEM at 37°C for 72 h, that is, the timeframe of the competition binding experiments. The results showed that compounds **4**–**7** were stable, whereas **2** and **3** exhibited degradation profiles, likely due to the hydrolysis of the trifluoroacetamide moiety (Figure S1). This is consistent with what was previously observed for compound **1**, which was hydrolysed in ~1 h [21]. The slower kinetics of **2**–**3** make them suitable for short‐time analysis but preclude analysis by real‐time in‐cell NMR. Therefore, compounds **1**–**3** were excluded from further analysis. CA1, CA2 and CA13 were selected as target CA isoforms for competitive binding experiments with fluorinated ligands **4**–**7**. Compound **6** was chosen as the spy ligand for real‐time in‐cell ^19^F NMR experiments, as it exhibited the fastest cellular uptake, an intermediate estimated affinity in the range of various compounds for both CA2 and CA13 isoforms, and no detectable off‐target interactions with other intracellular components [21]. Compounds **4**, **5** and **7** were employed as test ligands in the competition assays.

**FIGURE 2:**
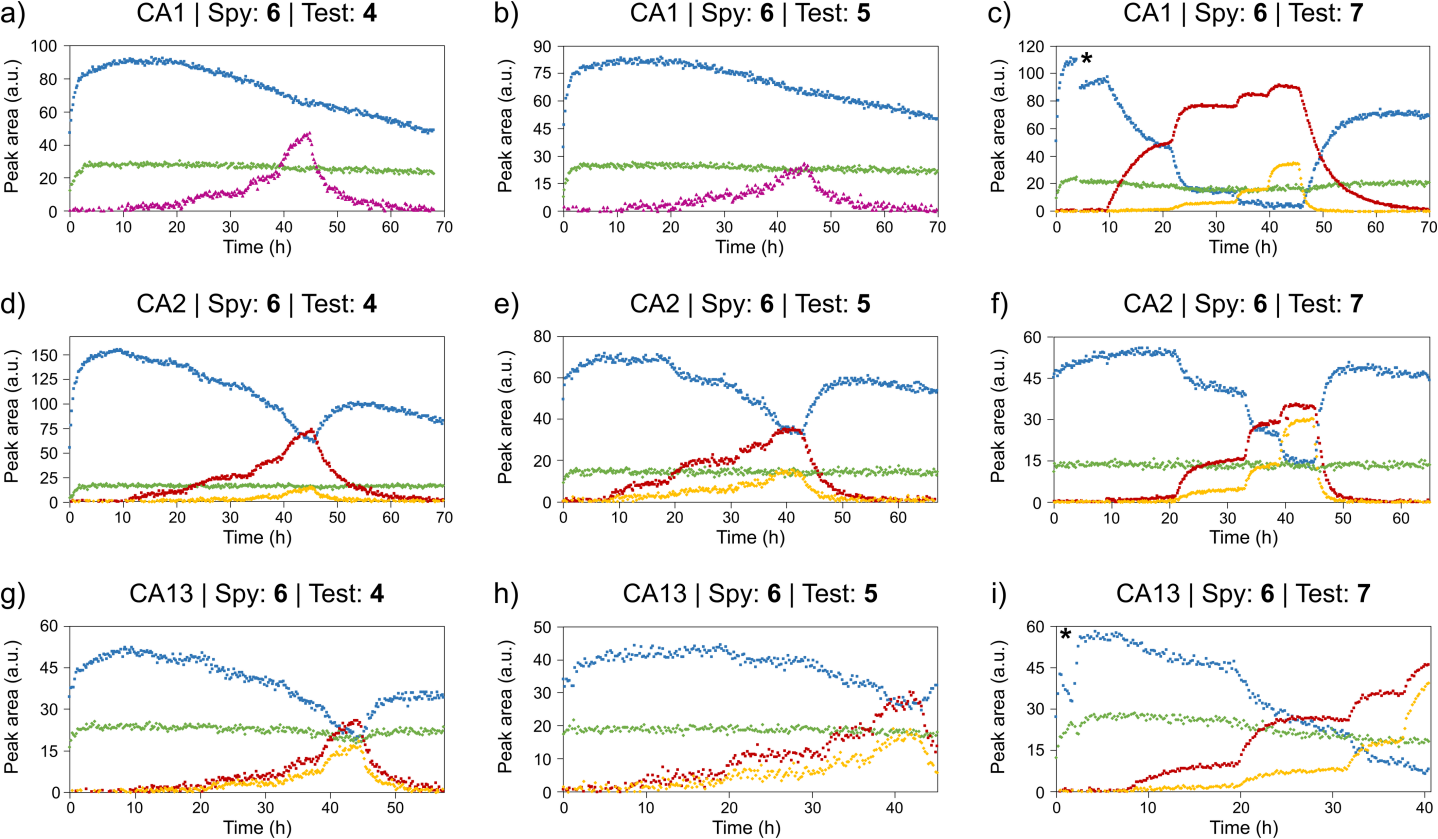
Time‐resolved in‐cell ^19^F NMR competition binding experiments. Competition assays were performed in the NMR bioreactor with compound **6** as the constant spy ligand and compounds **4** (a,d,g), **5** (b,e,h) and **7** (c,f,i) as test ligands, targeting CA1 (a–c), CA2 (d–f) and CA13 (g–i). Peak areas corresponding to free compound **6** (green), CA:**6** (blue), free test compound (yellow) and CA:Test (red) are plotted as a function of time. In (a) and (b), the free and bound signals of the test compound could not be separated and were therefore treated as a single combined signal (magenta). Theoretical concentrations of compounds **6** and test ligands at each step of the bioreactor runs are reported in Table S1. A discontinuity in the peak areas observed in (c) and (i), indicated with *, was caused by the presence of a gas bubble in the flow tube, which required a temporary interruption of the flow to remove it before resuming the experiment. [Colour figure can be viewed at wileyonlinelibrary.com]

Competitive binding experiments were conducted using real‐time ^19^F NMR on cells expressing CA1, CA2 or CA13, with a constant concentration of compound **6** and gradually increasing the concentration of the test ligand (Table S1). In each experiment, the displacement of compound **6** was monitored as the test ligand engaged the target CA isoform. This was reflected by a progressive decrease in the ^19^F NMR signal corresponding to the CA–compound **6** complex (CA:**6**) and by an increase in the signal corresponding to the CA–test ligand complex (CA:Test) (Figures 2 and S2). In comparison, no displacement of compound **6** was observed in two control experiments on cells expressing CA2, where DMSO was provided in place of the test ligand (Figure S3). In contrast to a previous real‐time in‐cell ^19^F NMR application, in which only the target–spy ligand complex was monitored, here the presence of fluorine on both ligands allowed simultaneous tracking of the two CA–ligand complexes, thereby providing a comprehensive view of the intracellular competitive binding. Notably, the resulting affinity measurements were independent of temporal variations in intracellular protein concentration over time. Furthermore, the signals of the free compounds were monitored in the competition binding experiments, except for compounds **4** and **5** competing with compound **6** for binding to CA1 (Figure 2a,b), where the signals of free and bound test ligand overlapped and could not be quantified separately. These signals were therefore treated as a single, combined signal.

Comparison of the signal intensity of the free spy ligand and the free test ligands revealed discrepancies between the observed ratios and what was expected from their concentration in the reservoirs. Therefore, to obtain a more accurate estimate of the ratio between free test ligand and free spy ligand, the peak areas of the corresponding signals were used. The resulting ratios (Table S2) deviated significantly from the theoretical values for compounds **4** and **5**, especially at higher concentrations. In contrast, compound **7** showed minimal deviation, with measured ratios closely matching those calculated from the concentrations in the reservoirs. The deviation observed for compounds **4** and **5** is likely attributable to their limited aqueous solubility, which is further supported by the low signal intensity observed in the stability measurements, whereas compounds **6** and **7** at the same concentration were instead fully soluble and resulted in higher signal intensities (Figure S1).

**FIGURE 3:**
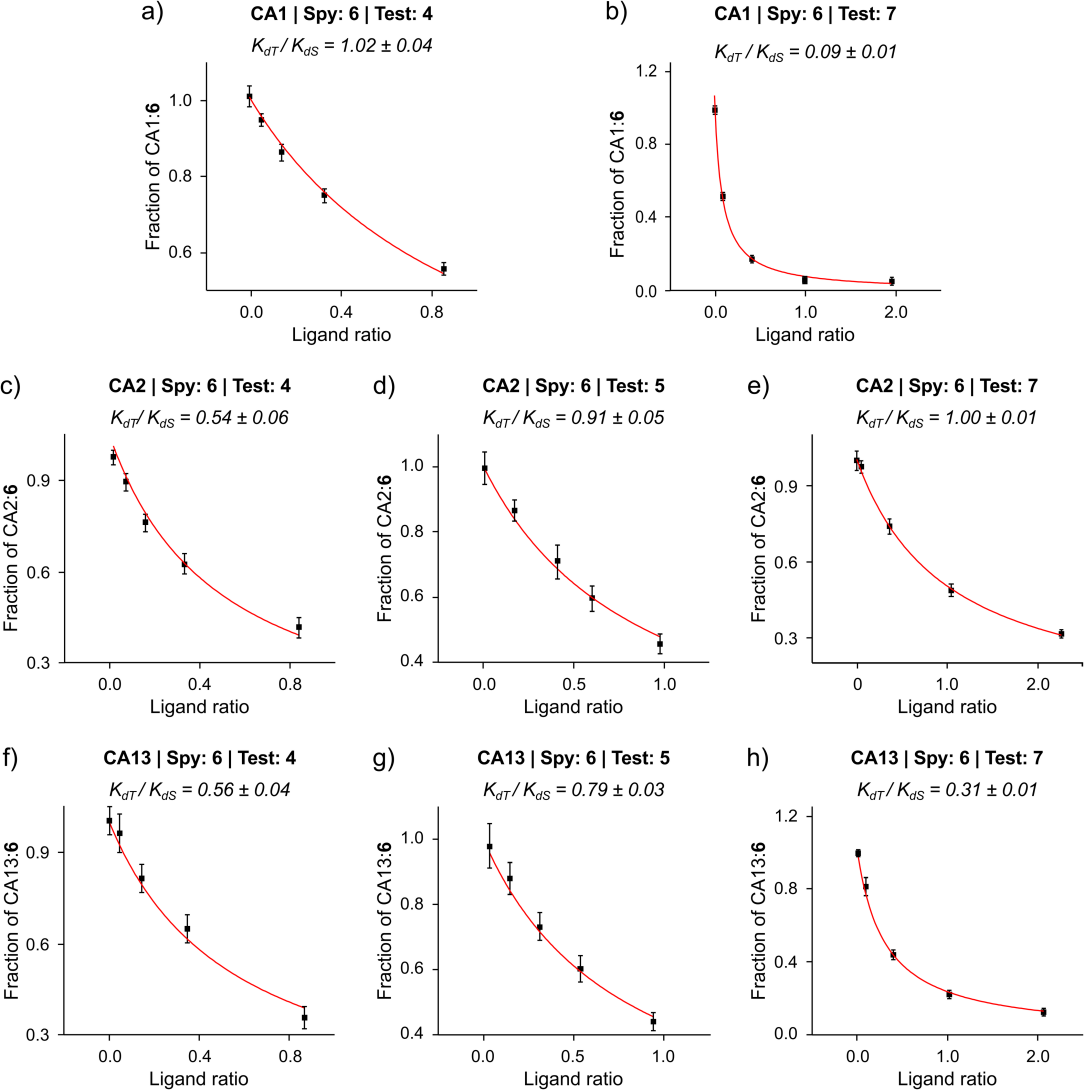
Fitting of competition binding data corrected for saturation effects for each CA–ligand complex. The plots show the fraction of CA1 (a,b), CA2 (c–e) and CA13 (f–h) bound to compound **6** at the end of each concentration step during the bioreactor runs in competition with compound **4** (a,c,f), **5** (d,g) and **7** (b,e,h). Data are plotted as a function of the ratio between the free concentrations of the test compound and the spy compound. Nonlinear regression fits are shown as red curves. The resulting intracellular *K*_*dT*_/*K*_*dS*_ values for each test compound are reported. [Colour figure can be viewed at wileyonlinelibrary.com]

When quantifying populations by signal integration, the partial saturation of the signals caused by a short repetition time can decrease the accuracy of the measurement if the ^19^F longitudinal relaxation time (*T*_1_) of the two signals differs. To account for signal saturation, intracellular ^19^F *T*_1_ of compounds **4**–**7** bound to each CA isoform was measured (Table S3), and the calculated SFs were used to correct the corresponding peak areas, from which the molar fraction of CA bound to compound **6** (CA:**6**) was obtained (see Section 2). For each competition binding experiment, the fraction of CA:**6** as a function of the ratios of free ligand concentrations was fitted with Equation (1) to determine the intracellular *K*_*dT*_/*K*_*dS*_ ratio (Figure 3). When the same fitting was performed using the uncorrected peak areas (Figure S4 and Table S4), some *K*_*dT*_/*K*_*dS*_ ratios deviated appreciably (up to 30%) from those obtained from the corrected fitting, indicating that saturation effects were indeed not negligible in these experimental conditions.

The resulting intracellular *K*_*dT*_/*K*_*dS*_ ratios ranged approximately from 0.09 to 1.02 for CA1, from 0.55 to 1.64 for CA2 and from 0.31 to 0.79 for CA13 (Table 1). Notably, in the competition experiment between compounds **5** and **6** targeting CA1 (Figure 2b), the signals of free compound **5** and CA1:**5** overlapped, with the complex signal appearing poorly resolved or entirely absent. Indeed, the similarity in intensity of free compounds **5** and **6** observed with the same ligands on different isoforms (Figure 2e,h) supports the conclusion that the formation of CA1:**5** was negligible and, consequently, the *K*_*dT*_/*K*_*dS*_ ratio is ≫1. In the competition between compounds **6** and **4** on CA1, the signals of free and bound **4** also overlapped, but in this case, the total signal intensity of compound **4** in the final step was clearly higher than that of free compound **6** (Figure 2a), indicating that the overlapping peak corresponded to both free **4** and the CA1:**4**. This overlap results in an overestimation of the CA1:**4** population and thus an underestimation of the corresponding *K*_*dT*_/*K*_*dS*_ ratio.

**TABLE 1:** Binding affinities relative to compound **6** derived from saturation‐corrected real‐time in‐cell ^19^F NMR data. For each experiment, the corrected *K*_*dT*_/*K*_*dS*_ values are reported.

Compound	*K*_*dT*_/*K*_*dS*_ for CA1	*K*_*I*_ (nM) for CA1^a^	*K*_*dT*_/*K*_*dS*_ for CA2	*K*_*I*_ (nM) for CA2^a^	*K*_*dT*_/*K*_*dS*_ for CA13	*K*_*I*_ (nM) for CA13^a^
**4**	> 1.02 ± 0.04	9.7	0.55 ± 0.06	1150	0.56 ± 0.04	153
**5**	n.d. (≫1)	142	0.91 ± 0.05	203	0.79 ± 0.03	82.0
**6** (spy)	1	49.3	1	78.0	1	230
**7**	0.09 ± 0.01	81.6	1.64 ± 0.06	57.6	0.31 ± 0.01	124

^a^ In vitro stopped‐flow assay [21].

**FIGURE 4:**
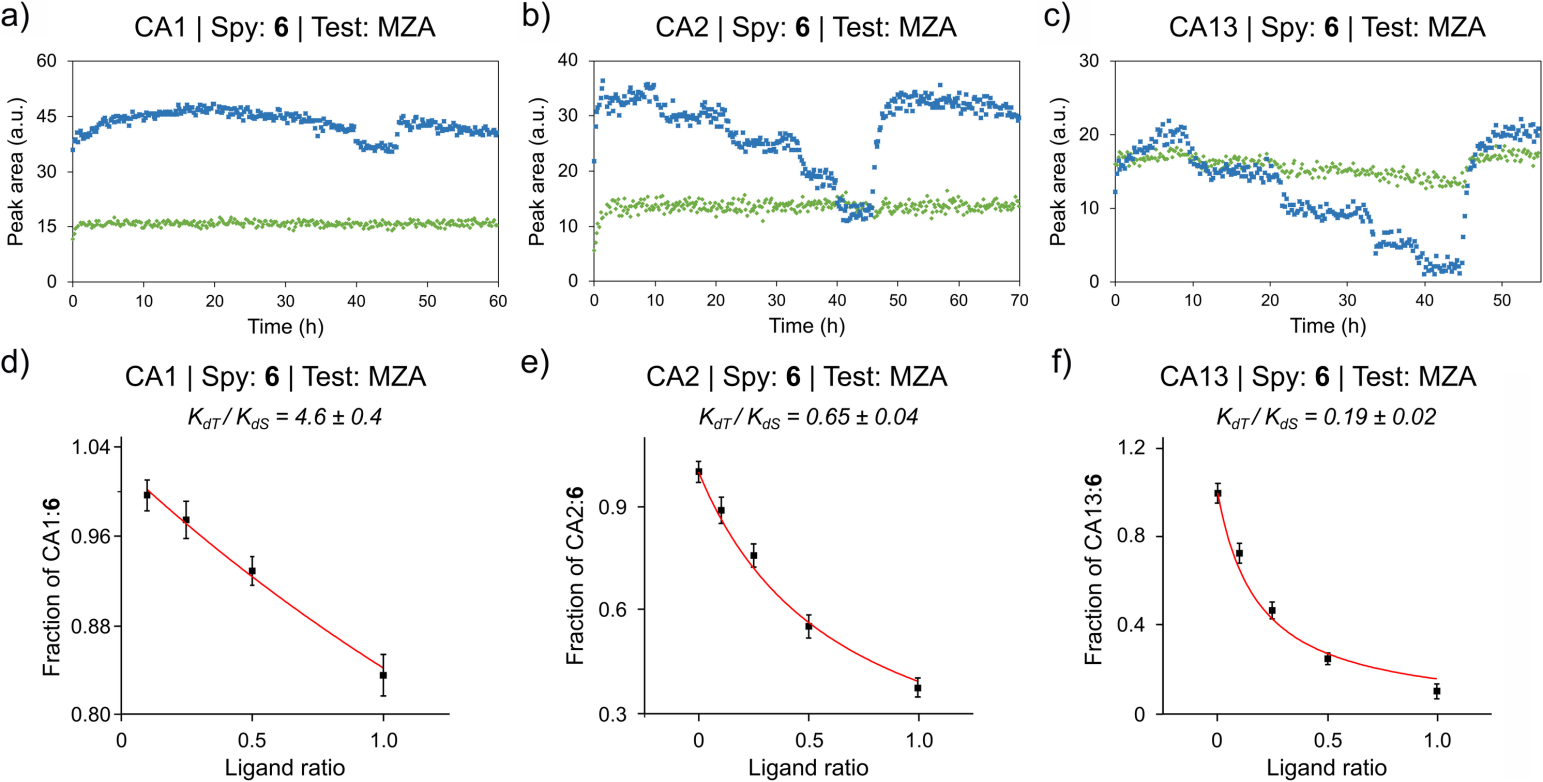
Time‐resolved in‐cell ^19^F NMR competition binding between compound **6** and methazolamide for each CA isoform. Competition assays (a–c) performed in the NMR bioreactor with compound **6** as the constant spy ligand and methazolamide (MZA), targeting CA1 (a), CA2 (b) and CA13 (c). Peak areas correspond to free compound **6** (green) and CA:**6** (blue). Concentrations of compounds **6** and test ligands at each step of the bioreactor runs are reported in Table S5. Plots (d–f) show the fraction of CA1 (d), CA2 (e) and CA13 (f) bound to compound **6** as a function of the ratio between the concentrations of MZA and compound **6** at the end of each step. Red curves represent nonlinear regression fits used to determine the intracellular *K*_*dT*_/*K*_*dS*_ values. [Colour figure can be viewed at wileyonlinelibrary.com]

To estimate the *K*_*d*_ values of these novel compounds, additional competition binding experiments were performed between the spy compound **6** and methazolamide, a well‐characterized CA inhibitor. Real‐time ^19^F NMR experiments were carried out on cells expressing CA1, CA2 or CA13 (Figure 4 and Table S5). The resulting *K*_*dT*_/*K*_*dS*_ ratios were used to calculate the corresponding intracellular *K*_*d*_ values (Figure 4). As *K*_*d*_ values for methazolamide are not reported for all isoforms, *K*_*I*_ values determined in vitro (50, 14 and 19 nM for CA1, CA2 and CA13, respectively) were used as reference [28]. The calculated dissociation constant (*K*_*d_calc*_) of compound **6** was first determined from its competition with methazolamide and then used to derive the *K*_*d_calc*_ values of the other ligands based on their *K*_*dT*_/*K*_*dS*_ ratios (Table 2).

**TABLE 2:** Calculated dissociation constant (*K*_*d_calc*_) values of compounds **4**–**7** for each CA isoform, derived from the *K*_*d*_ ratios obtained in the real‐time in‐cell ^19^F NMR competition binding experiments.

Compound	*K*_*d_calc*_ for CA1 (nM)	*K*_*d_calc*_ for CA2 (nM)	*K*_*d_calc*_ for CA13 (nM)
**4**	11 ± 2	12 ± 3	57 ± 16
**5**	n.d.	19 ± 4	80 ± 20
**6**	11 ± 2	22 ± 3	101 ± 21
**7**	1.0 ± 0.3	35 ± 7	32 ± 8

Overall, all compounds exhibited good affinity towards the three CA isoforms, except for compound **5**, which showed markedly reduced affinity for CA1. Interestingly, compound **7** displayed higher affinity for CA1, suggesting enhanced selectivity for this isoform compared with CA2 and CA13. Furthermore, the *K*_*d_calc*_ values for CA1 and CA13 were in agreement with the qualitative affinity ranking previously reported [21], whereas for CA2, compound **7** appeared weaker than predicted, likely due to the fact that all compounds displayed similar *K*_*d_calc*_ values.

## 4 Conclusions

In this study, we applied real‐time in‐cell ^19^F NMR analysis to quantitatively assess competitive binding among fluorinated benzenesulfonamides targeting three cytosolic CA isoforms in human cells, using a ligand‐observed approach. Once a suitable ligand was chosen as spy, competition binding experiments with the other ligands were conducted to determine the intracellular *K*_*d*_ ratio between the test ligand and the spy ligand, thus providing a relative ranking of the fluorinated ligands based on their intracellular binding affinities for each CA isoform. The resulting data indicated that most compounds have overall comparable binding affinities across all isoforms, with the exception of compound **5**, which showed the lowest affinity, and compound **7**, which displayed the highest affinity for the CA1 isoform.

Unlike a previous application of real‐time in‐cell ^19^F NMR, in which only one ligand was monitored [14], competition binding between two fluorinated ligands allows tracking both ligands simultaneously. The observation of both ligand–target complexes allows quantifying the total amount of intracellular over the course of the bioreactor run. Doing so revealed that all three CA isoforms were slowly depleted from the cell sample, which may be caused by the intracellular proteins slowly being metabolized, leaking from the cells, or by a gradual release of cells from the agarose gel that are then removed by the flow of medium. Accurate analysis is therefore ensured regardless of changes in target protein levels over time. Moreover, the simultaneous detection of the free fluorinated ligands enables the experimental determination of their real concentration ratio in the sample. This is especially relevant in cases where one ligand, such as compound **4** or **5** in this study, exhibits limited aqueous solubility compared with the other. A non‐fluorinated test ligand—such as methazolamide—can still be analysed if needed, provided that the compound is sufficiently soluble. We also show that *T*_1_‐related differential signal saturation cannot be ignored when short repetition times are used but can be easily corrected a posteriori by measuring the *T*_1_ of the target–ligand complexes.

Concerning the development of future isoform‐selective CA inhibitors, the identification of compound **6** as a suitable spy ligand paves the way for applying this approach to assay other ligands by intracellular competitive binding assays, thus expanding the applicability of the method beyond the current set of fluorinated benzenesulfonamides. Furthermore, since compound **6** forms distinct ^19^F NMR chemical shifts when bound to different CA isoforms, this could in principle enable simultaneous competition binding experiments across multiple CA isoforms within the same cell sample. Indeed, provided that the ^19^F signals of the spy ligand in complex with different targets are sufficiently resolved, it should be possible to perform multiplexed assays where two test ligands are evaluated against multiple intracellular targets in parallel. Such an approach would offer significant advantages in terms of experimental efficiency and resource optimization.

## Funding

This work was supported by European Commission (101094131), European Union ‐ Next Generation EU (IR0000009) and Ministero dell'Università e della Ricerca (2022WANFH5).

## Conflicts of Interest

The authors declare no conflicts of interest.

## Supporting information


**Figure S1:** Stability tests of fluorinated compounds **1**–**7**.
**Figure S2:** Representative real‐time competition binding NMR experiment.
**Figure S3:** Control time‐resolved NMR experiments with DMSO.
**Figure S4:** Fitting of competition binding data before saturation factor correction.
**Table S1:** Experimental details of the bioreactor runs.
**Table S2:** Calculated free ligand concentration ratios.
**Table S3:** Longitudinal relaxation times and saturation factors.
**Table S4:** Affinity constants derived from real‐time in‐cell ^19^F NMR data.

## Data Availability

The data that support the findings of this study are available from the corresponding author upon reasonable request.
