# Supplementary material for: Quantitative Competition Binding of Fluorinated Ligands by Real‐Time 19F In‐Cell NMR to Assess Isoform Selectivity in Human Cells
Source: Magn Reson Chem. 2026 Mar 22;64(6):584–93. doi: 10.1002/mrc.70098 (PMC13135872; doi:10.1002/mrc.70098)
Supplement: Supplementary file 1 — Figure S1: Stability tests of fluorinated compounds 1–7. Figure S2: Representative real‐time competition binding NMR experiment. Figure S3: Control time‐resolved NMR experiments with DMSO. Figure S4: Fitting of competition binding data before saturation factor correction. Table S1: Experimental details of the bioreactor runs. Table S2: Calculated free ligand concentration ratios. Table S3: Longitudinal relaxation times and saturation factors. Table S4: Affinity constants derived from real‐time in‐cell 19F NMR data. [file MRC-64-584-s001.docx]

**Supporting Information**

**Quantitative competition binding of fluorinated ligands by real-time ^19^F in-cell NMR to assess isoform selectivity in human cells**

Azzurra Costantino^a^, Letizia Barbieri^a,b^, Simone Giovannuzzi^c^, Alessio Nocentini^c^, Claudiu T. Supuran^c^, Enrico Luchinat^a,b,d^*

^a^ Magnetic Resonance Center – CERM, University of Florence, via Luigi Sacconi, 6, 50019, Sesto Fiorentino, Italy;

^b^ Interuniversity Consortium for Magnetic Resonance of Metalloproteins – CIRMMP, via Luigi Sacconi, 6, 50019, Sesto Fiorentino, Italy;

^c^ NEUROFARBA Department, Section of Pharmaceutical and Nutraceutical Sciences, University of Florence, Sesto Fiorentino, Italy;

^d^ Chemistry Department, University of Florence, Via della Lastruccia, 3, 50019, Sesto Fiorentino, Italy.


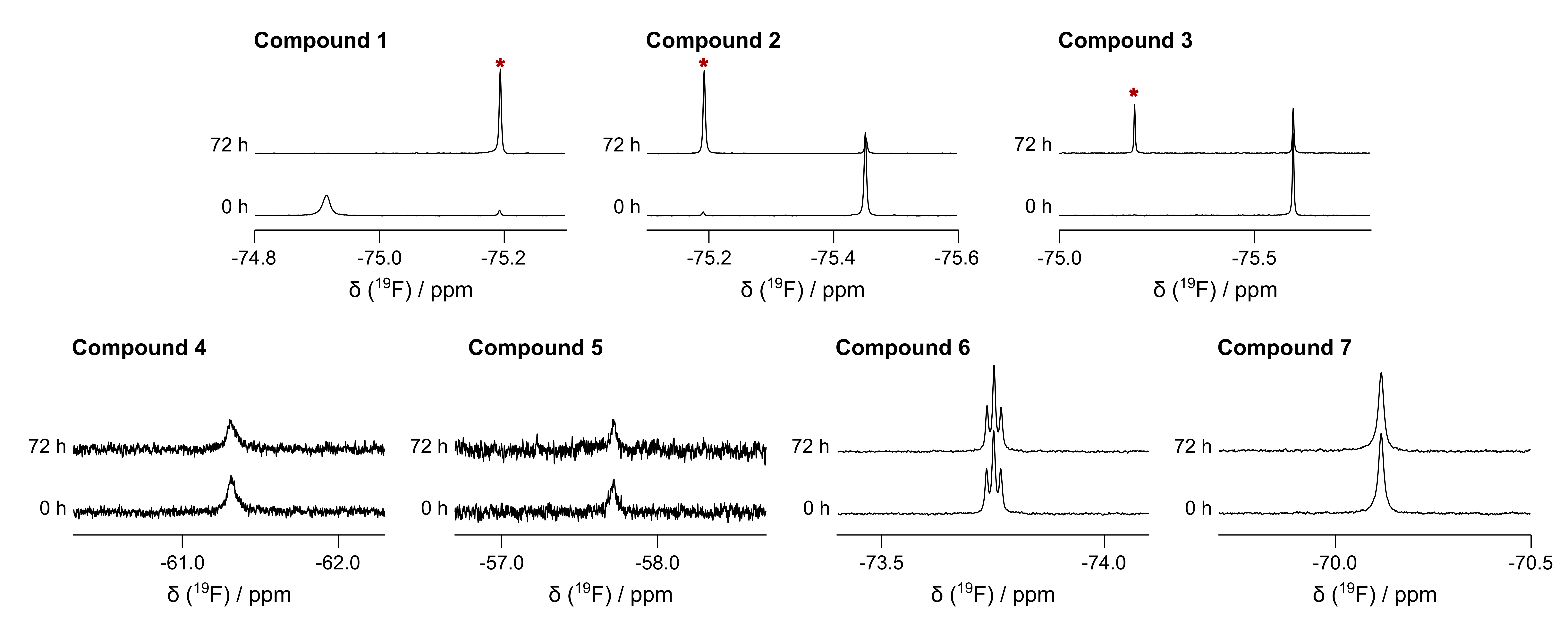


**Figure S1:** Stability tests of fluorinated compounds in DMEM at 37°C. ^19^F NMR spectra of compounds **1**–**7** dissolved in DMEM (supplemented with 1% antibiotics, 2% FBS, and 3% D₂O), acquired at time zero and after 72 hours at 37°C to assess ligand stability in cell culture medium. The intensities of the original compound peak and the degradation product (indicated by *) were tracked. The same degradation product was observed for compounds **1**-**3**, thus suggesting that the trifluoroacetamide group present in these three compounds is hydrolysed to produce trifluoroacetate.


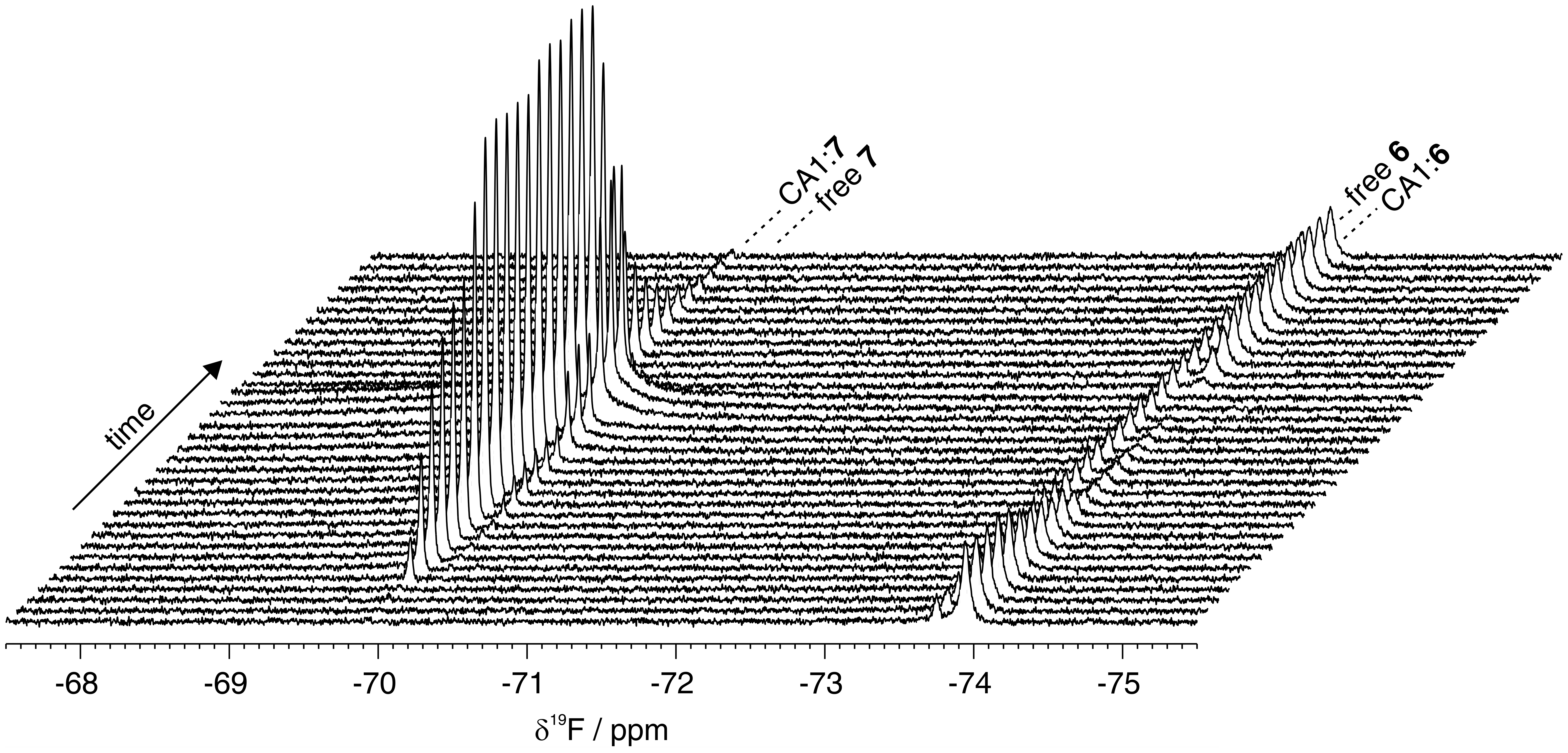


**Figure S2:** Representative real-time competition binding NMR experiment. Waterfall plot of 1D ^19^F NMR spectra recorded on cells expressing CA1, with compound **6** as the spy ligand and compound **7** as test ligand, recorded over the course of ~70 hours (see **Figure 2c**). For clarity, only every 10th NMR spectrum is shown. The signals arising from free and CA1-bound compounds are labelled accordingly.


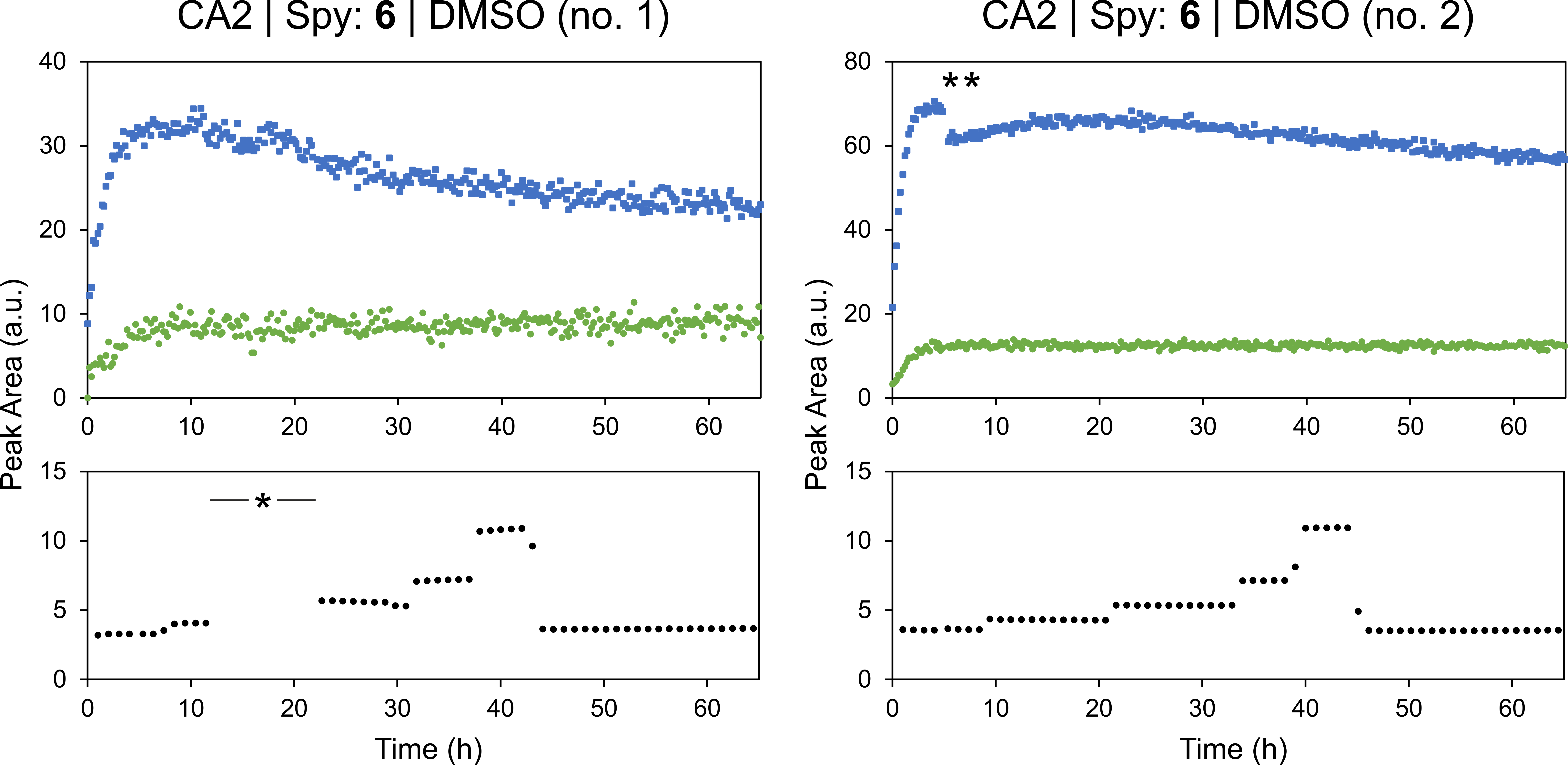


**Figure S3.** Control time-resolved NMR experiments with increasing concentration of DMSO. Two bioreactor runs were performed in the NMR bioreactor with compound **6** as the constant spy ligand and DMSO alone in place of the test ligand. In the top panels, ^19^F peak areas corresponding to free compound **6** (green) and CA:**6** (blue) are plotted as a function of time. In the bottom panels, the ^1^H peak area corresponding to DMSO (black) is plotted as a function of time. Theoretical concentrations of compounds **6** and DMSO at each step of the bioreactor runs are reported in **Table S1**. The ^1^H and ^19^F intensities are not comparable to one another. * the lack of data for DMSO is due to a loss of field homogeneity occurring overnight, which did not affect the integration of the ^19^F peaks but prevented the analysis of the water-suppressed ^1^H NMR spectra. ** the discontinuity in the peak areas was caused by the presence of a gas bubble in the flow tube, which required a temporary interruption of the flow to remove it before resuming the experiment.


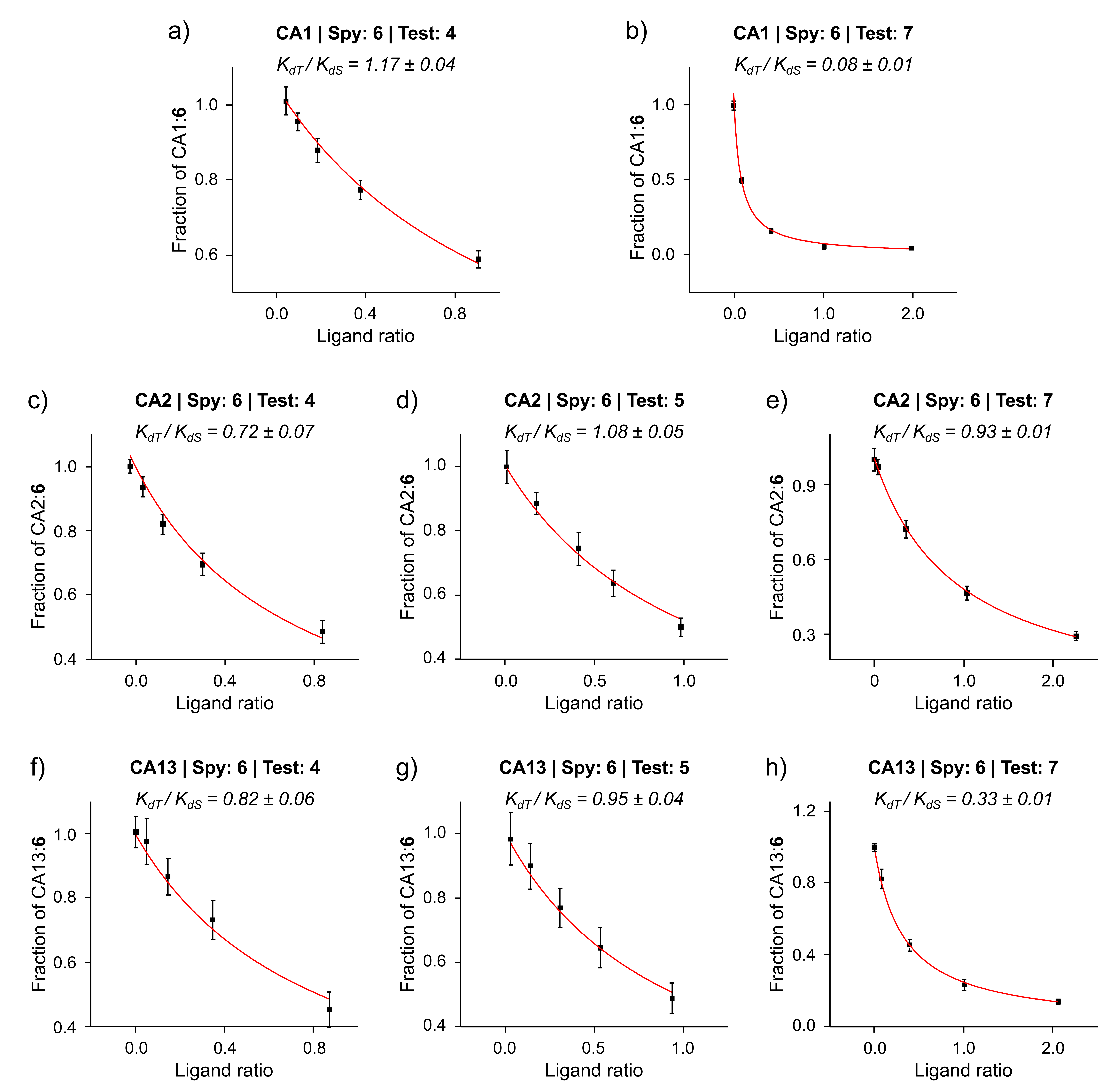


**Figure S4.** Fitting of competition binding data before saturation factor correction. Plots show the fraction of CA1 (a,b), CA2 (c–e), and CA13 (f-h) isoforms bound to compound **6** at the end of each concentration step during the bioreactor runs, plotted as a function of the ratio between the free concentrations of the test compounds **4** (a,c,f), **5** (d,g) and **7** (b,e,h) and the spy compound **6**. Nonlinear regression fits are shown as red curves. The resulting intracellular K_dT_/K_dS_ values for each test compound are reported in the corresponding panels.

**Table S1.** Durations and ligand concentrations of each step of the bioreactor runs reported in Figure 2 and Figure S3. For each channel, ligand concentrations in the reservoir and flow rates are also reported. In the control runs, the concentration of DMSO is reported as % (v/v).

| **Bioreactor run 1 (Figure 2a)** – Target: CA1 – *Channel 1:* **6** (10 µM); *Channel 2:* **6** (10 µM) + **4** (20 µM) | | | | | |
| --- | --- | --- | --- | --- | --- |
| **Step** | **Duration (h)** | **6 (µM)** | **4 (µM)** | **Channel 1 flow rate (µL/min)** | **Channel 2 flow rate (µL/min)** |
| 1 | 8 | 10 | 0 | 100 | 0 |
| 2 | 12 | 10 | 2 | 90 | 10 |
| 3 | 12 | 10 | 5 | 75 | 25 |
| 4 | 6 | 10 | 10 | 50 | 50 |
| 5 | 6 | 10 | 20 | 0 | 100 |
| **Bioreactor run 2 (Figure 2b)** – Target: CA1 – *Channel 1:* **6** (10 µM); *Channel 2:* **6** (10 µM) + **5** (20 µM) | | | | | |
| **Step** | **Duration (h)** | **6 (µM)** | **5 (µM)** | **Channel 1 flow rate (µL/min)** | **Channel 2 flow rate (µL/min)** |
| 1 | 7 | 10 | 0 | 100 | 0 |
| 2 | 12 | 10 | 2 | 90 | 10 |
| 3 | 12 | 10 | 5 | 75 | 25 |
| 4 | 6 | 10 | 10 | 50 | 50 |
| 5 | 6 | 10 | 20 | 0 | 100 |
| **Bioreactor run 3 (Figure 2c)** – Target: CA1 – *Channel 1:* **6** (10 µM); *Channel 2:* **6** (10 µM) + **7** (20 µM) | | | | | |
| **Step** | **Duration (h)** | **6 (µM)** | **7 (µM)** | **Channel 1 flow rate (µL/min)** | **Channel 2 flow rate (µL/min)** |
| 1 | 9 | 10 | 0 | 100 | 0 |
| 2 | 12 | 10 | 2 | 90 | 10 |
| 3 | 12 | 10 | 5 | 75 | 25 |
| 4 | 6 | 10 | 10 | 50 | 50 |
| 5 | 6 | 10 | 20 | 0 | 100 |
| **Bioreactor run 4 (Figure 2d)** – Target: CA2 – *Channel 1:* **6** (10 µM); *Channel 2:* **6** (10 µM) + **4** (20 µM) | | | | | |
| **Step** | **Duration (h)** | **6 (µM)** | **4 (µM)** | **Channel 1 flow rate (µL/min)** | **Channel 2 flow rate (µL/min)** |
| 1 | 9 | 10 | 0 | 100 | 0 |
| 2 | 12 | 10 | 2 | 90 | 10 |
| 3 | 12 | 10 | 5 | 75 | 25 |
| 4 | 6 | 10 | 10 | 50 | 50 |
| 5 | 6 | 10 | 20 | 0 | 100 |
| **Bioreactor run 5 (Figure 2e)** – Target: CA2 – *Channel 1:* **6** (10 µM); *Channel 2:* **6** (10 µM) + **5** (20 µM) | | | | | |
| **Step** | **Duration (h)** | **6 (µM)** | **5 (µM)** | **Channel 1 flow rate (µL/min)** | **Channel 2 flow rate (µL/min)** |
| 1 | 6 | 10 | 0 | 100 | 0 |
| 2 | 12 | 10 | 2 | 90 | 10 |
| 3 | 12 | 10 | 5 | 75 | 25 |
| 4 | 6 | 10 | 10 | 50 | 50 |
| 5 | 6 | 10 | 20 | 0 | 100 |
| **Bioreactor run 6 (Figure 2f)** – Target: CA2 – *Channel 1:* **6** (10 µM); *Channel 2:* **7** (20 µM) | | | | | |
| **Step** | **Duration (h)** | **6 (µM)** | **7 (µM)** | **Channel 1 flow rate (µL/min)** | **Channel 2 flow rate (µL/min)** |
| 1 | 7 | 10 | 0 | 100 | 0 |
| 2 | 12 | 9 | 2 | 90 | 10 |
| 3 | 12 | 7,5 | 5 | 75 | 25 |
| 4 | 6 | 5 | 10 | 50 | 50 |
| 5 | 6 | 0 | 20 | 0 | 100 |
| **Bioreactor run 7 (Figure 2g)** – Target: CA13 **-** *Channel 1:* **6** (10 µM); *Channel 2:* **6** (10 µM) + **4** (20 µM) | | | | | |
| **Step** | **Duration (h)** | **6 (µM)** | **4 (µM)** | **Channel 1 flow rate (µL/min)** | **Channel 2 flow rate (µL/min)** |
| 1 | 9 | 10 | 0 | 100 | 0 |
| 2 | 12 | 10 | 2 | 90 | 10 |
| 3 | 12 | 10 | 5 | 75 | 25 |
| 4 | 6 | 10 | 10 | 50 | 50 |
| 5 | 6 | 10 | 20 | 0 | 100 |
| **Bioreactor run 8 (Figure 2h)** – Target: CA13 **-** *Channel 1:* **6** (10 µM); *Channel 2:* **6** (10 µM) + **5** (20 µM) | | | | | |
| **Step** | **Duration (h)** | **6 (µM)** | **5 (µM)** | **Channel 1 flow rate (µL/min)** | **Channel 2 flow rate (µL/min)** |
| 1 | 8 | 10 | 0 | 100 | 0 |
| 2 | 12 | 10 | 2 | 90 | 10 |
| 3 | 12 | 10 | 5 | 75 | 25 |
| 4 | 6 | 10 | 10 | 50 | 50 |
| 5 | 6 | 10 | 20 | 0 | 100 |
| **Bioreactor run 9 (Figure 2i)** – Target: CA13 – *Channel 1:* **6** (10 µM); *Channel 2:* **6** (10 µM) + **7** (20 µM) | | | | | |
| **Step** | **Duration (h)** | **6 (µM)** | **7 (µM)** | **Channel 1 flow rate (µL/min)** | **Channel 2 flow rate (µL/min)** |
| 1 | 7 | 10 | 0 | 100 | 0 |
| 2 | 12 | 10 | 2 | 90 | 10 |
| 3 | 12 | 10 | 5 | 75 | 25 |
| 4 | 6 | 10 | 10 | 50 | 50 |
| 5 | 6 | 10 | 20 | 0 | 100 |
| **Control runs (Figure S3)** – Target: CA2 – *Channel 1:* **6** (10 µM), DMSO (0.0125 %); *Channel 2:* **6** (10 µM), DMSO (0.0375 %) | | | | | |
| **Step** | **Duration (h)** | **6 (µM)** | **DMSO (%)** | **Channel 1 flow rate (µL/min)** | **Channel 2 flow rate (µL/min)** |
| 1 | 8 | 10 | 0.0125 | 100 | 0 |
| 2 | 12 | 10 | 0.015 | 90 | 10 |
| 3 | 12 | 10 | 0.01875 | 75 | 25 |
| 4 | 6 | 10 | 0.025 | 50 | 50 |
| 5 | 6 | 10 | 0.0375 | 0 | 100 |

**Table S2.** Free ligand concentration ratios between the test ligand and the spy ligand, calculated at the plateau of each concentration step (averaged over a 1-hour interval). The corresponding theoretical ratios, based on the nominal concentrations in the reservoir, are also reported for comparison.

| **Bioreactor runs 1, 2, and 3 with CA1 (Figure 2a, b, c)** | | | | |
| --- | --- | --- | --- | --- |
| **Step n°** | **Theoretical ratio [T]/[S]** | **Calculated [4]/[6] for CA1 *** | **Calculated [5]/[6] for CA1 **** | **Calculated [7]/[6] for CA1** |
| 1 | 0 | -0,01 | 0,02 | -0,01 |
| 2 | 0.2 | 0,04 | 0,16 | 0,08 |
| 3 | 0.5 | 0,14 | 0,36 | 0,41 |
| 4 | 1 | 0,32 | 0,57 | 1,01 |
| 5 | 2 | 0,85 | 0,96 | 1,98 |
| **Bioreactor runs 4, 5, and 6 with CA2 (Figure 2d, e, f)** | | | | |
| **Step n°** | **Theoretical ratio [T]/[S]** | **Calculated [4]/[6] for CA2** | **Calculated [5]/[6] for CA2** | **Calculated [7]/[6] for CA2** |
| 1 | 0 | -0,02 | 0,01 | 0,00 |
| 2 | 0.2 | 0,04 | 0,17 | 0,04 |
| 3 | 0.5 | 0,12 | 0,41 | 0,35 |
| 4 | 1 | 0,30 | 0,61 | 1,04 |
| 5 | 2 | 0,83 | 0,98 | 2,26 |
| **Bioreactor runs 7, 8, and 9 with CA1 (Figure 2g, h, i)** | | | | |
| **Step n°** | **Theoretical ratio [T]/[S]** | **Calculated [4]/[6] for CA13** | **Calculated [5]/[6] for CA13** | **Calculated [7]/[6] for CA13** |
| 1 | 0 | 0 | 0,03 | 0,01 |
| 2 | 0.2 | 0,05 | 0,14 | 0,09 |
| 3 | 0.5 | 0,15 | 0,31 | 0,40 |
| 4 | 1 | 0,35 | 0,53 | 1,01 |
| 5 | 2 | 0,87 | 0,93 | 2,06 |

** Values calculated from the average [4]/[6] ratios for CA2 and CA13.*

*** Values calculated from the average [5]/[6] ratios for CA2 and CA13.*

**Table S3.** Longitudinal relaxation times (T_1_) and corresponding saturation factors (SF) for each CA–ligand complex. T_1_ values were determined using the ‘Inversion Recovery with partial inversion’ function in Dynamics Center, and the associated saturation factors were calculated using Equation 2. These SF values were subsequently used to correct the peak areas of the CA–ligand complexes prior to nonlinear fitting and determination of the K_dT_/K_dS_ ratios.

| **T_1_ and SF for CA1-compound complex** | | |
| --- | --- | --- |
| **Compound** | **T_1_ (s)** | **SF** |
| **4** | 1.26 ± 0.13 | 0.64 ± 0.04 |
| **5** | 1.00 ± 0.06 | 0.73 ± 0.02 |
| **6** | 0.98 ± 0.09 | 0.73 ± 0.03 |
| **7** | 0.78 ± 0.02 | 0.81 ± 0.01 |
| **T_1_ and SF for CA2-compound complex** | | |
| **Compound** | **T_1_ (s)** | **SF** |
| **4** | 1.30 ± 0.15 | 0.64 ± 0.04 |
| **5** | 1.07 ± 0.09 | 0.70 ± 0.03 |
| **6** | 0.73 ± 0.02 | 0.83 ± 0.01 |
| **7** | 0.55 ± 0.01 | 0.91 ± 0.01 |
| **T_1_ and SF for CA13-compound complex** | | |
| **Compound** | **T_1_ (s)** | **SF** |
| **4** | 1.30 ± 0.12 | 0.64 ± 0.04 |
| **5** | 0.87 ± 0.04 | 0.77 ± 0.01 |
| **6** | 0.48 ± 0.03 | 0.93 ± 0.01 |
| **7** | 0.61 ± 0.03 | 0.88 ± 0.01 |

**Table S4**. Affinity Constants for CA1, CA2, and CA13 derived from Real-Time In-Cell ^19^F NMR Data.

| **Compound** | **K_dT_/K_dS_ for CA1 by in-cell NMR** | **Ki (nM) for CA1^a^** | **K_dT_/K_dS_ for CA2 by in-cell NMR** | **Ki (nM) for CA2^a^** | **K_dT_/K_dS_ for CA13 by in-cell NMR** | **Ki (nM) for CA13^a^** |
| --- | --- | --- | --- | --- | --- | --- |
| **4** | 1.17 ± 0.04 | 9.7 | 0.72 ± 0.07 | 1150 | 0.82 ± 0.06 | 153 |
| **5** | >>1 | 142 | 1.08 ± 0.05 | 203 | 0.95 ± 0.04 | 82.0 |
| **6** (spy) | 1 | 49.3 | 1 | 78.0 | 1 | 230 |
| **7** | 0.08 ± 0.01 | 81.6 | 1.51± 0.05 | 57.6 | 0.33 ± 0.01 | 124 |

*^a^Ki taken from Costantino et al.*^[1]^

**Table S5.** Durations and ligand concentrations of each step of the bioreactor runs for competition binding experiments between compound 6 and methazolamide reported in Figure S3. For each channel, ligand concentrations in the reservoir and flow rates are also reported.

| **Bioreactor run 10 (Figure S3a)** – Target: CA1 – *Channel 1:* **6** (10 µM); *Channel 2:* **6** (10 µM) + MZA (10 µM) | | | | |
| --- | --- | --- | --- | --- |
| **Duration (h)** | **6 (µM)** | **MZA (µM)** | **Channel 1 flow rate (µL/min)** | **Channel 2 flow rate (µL/min)** |
| 8 | 10 | 0 | 100 | 0 |
| 12 | 10 | 1 | 90 | 10 |
| 12 | 10 | 2.5 | 75 | 25 |
| 6 | 10 | 5 | 50 | 50 |
| 6 | 10 | 10 | 0 | 100 |
| **Bioreactor run 11 (Figure S3b)** – Target: CA2 – *Channel 1:* **6** (10 µM); *Channel 2:* **6** (10 µM) + MZA (10 µM) | | | | |
| **Duration (h)** | **6 (µM)** | **MZA (µM)** | **Channel 1 flow rate (µL/min)** | **Channel 2 flow rate (µL/min)** |
| 7 | 10 | 0 | 100 | 0 |
| 12 | 10 | 2 | 90 | 10 |
| 12 | 10 | 5 | 75 | 25 |
| 6 | 10 | 10 | 50 | 50 |
| 6 | 10 | 20 | 0 | 100 |
| **Bioreactor run 12 (Figure S3c)** – Target: CA13 – *Channel 1:* **6** (10 µM); *Channel 2:* **6** (10 µM) + MZA (10 µM) | | | | |
| **Duration (h)** | **6 (µM)** | **MZA (µM)** | **Channel 1 flow rate (µL/min)** | **Channel 2 flow rate (µL/min)** |
| 9 | 10 | 0 | 100 | 0 |
| 12 | 10 | 2 | 90 | 10 |
| 12 | 10 | 5 | 75 | 25 |
| 6 | 10 | 10 | 50 | 50 |
| 6 | 10 | 20 | 0 | 100 |

**References**

[1] A. Costantino, L. Barbieri, S. Giovannuzzi, A. Nocentini, C. T. Supuran, M. Raitelaitis, P. Nordlund, L. Banci, E. Luchinat, “Intracellular Binding of Novel Fluorinated Compounds to Carbonic Anhydrase Isoforms Explored by In-Cell 19F NMR” *J. Med. Chem.* **2025**, *68*, 23363–23374.
